# Lithium Toxicity or Alcohol Withdrawal?

**DOI:** 10.7759/cureus.110838

**Published:** 2026-06-14

**Authors:** Tanya Nagpal, Taylore Baker, Audrey S Farooqui

**Affiliations:** 1 Psychiatry, University of Louisville School of Medicine, Louisville, USA

**Keywords:** alcohol use disorder (aud), alcohol withdrawal syndrome, bipolar i disorder, hemodialysis, lithium, lithium toxicity

## Abstract

Lithium has a narrow therapeutic index and remains the first-line treatment for bipolar I and II disorders. Dosing this medication requires careful monitoring, and there is a risk of toxicity. This case follows a 43-year-old male with a medical history of alcohol use and bipolar I disorder, admitted to the hospital three days after an intentional lithium overdose. Initial management involved treating alcohol withdrawal with scheduled phenobarbital, lorazepam, and intravenous fluids. In the following days, he demonstrated a neurologic decline in the setting of subtherapeutic serum lithium levels. As the patient continued to worsen, hemodialysis was eventually initiated due to concern for lithium neurotoxicity. After completing three rounds of hemodialysis, the patient subsequently returned to a normal neurologic baseline. This case highlights the significance of clinical manifestations of lithium toxicity in the setting of subtherapeutic and down-trending serum lithium to manage lithium toxicity. Pre-existing tissue stores and rate of elimination contribute to the discrepancy between the severity of symptoms and serum levels in those with chronic lithium use.

## Introduction

Lithium has been used as a psychiatric treatment since the mid-twentieth century, most notably following Dr. John Cade’s 1949 reports of its efficacy in mania, and it remains the first-line therapy for bipolar I and II disorders today [1]. Although the precise mechanism of action of lithium is not fully understood, it is thought to modulate several intracellular signaling pathways, including inhibition of glycogen synthase kinase-3 and inositol monophosphatase, which are implicated in mood regulation [2]. Clinically available formulations include lithium carbonate and lithium citrate, both of which are renally excreted. Lithium has a narrow therapeutic window of 0.8-1.2 mEq/L and a long half-life of approximately 18-36 hours, which is prolonged further with chronic use as tissue compartments become saturated [1,2]. Because lithium distributes slowly into the central nervous system (CNS) via passive diffusion and accumulates in tissues with chronic use, serum levels do not always reflect the degree of intracellular or CNS exposure. This discordance between serum lithium concentration and clinical severity is the central diagnostic challenge illustrated in this report. Lithium toxicity can produce CNS complications including delirium, new or worsening tremors, clonus, hyperreflexia, seizures, respiratory depression, and coma [2]. Additional adverse effects include bradycardia, sick sinus syndrome, goiter, nephrogenic diabetes insipidus (a condition in which the kidneys lose the ability to concentrate urine despite adequate antidiuretic hormone), and aplastic anemia [2]. Treatment of lithium toxicity begins with immediate discontinuation of the drug and IV fluid administration to promote renal excretion. In severe cases, extracorporeal treatment (specifically hemodialysis, a process that filters lithium directly from the blood) is required [3]. Standard criteria for initiating hemodialysis include very high serum lithium levels (typically above 4.0 mEq/L) and signs of neurotoxicity [3]. Several case reports suggest that serum lithium levels may not reliably predict toxicity severity. One case involved an 18-year-old female patient newly initiated on lithium who was admitted medically for hyperreflexia, myoclonus, rigidity, slurred speech, anuria, facial edema, and disorientation. Her lithium levels were within the therapeutic range (0.8-1.2 mEq/L), and she was treated with IV mannitol to aid in excretion of lithium with recovery within one week [3]. Another case described a 45-year-old female patient prescribed 900mg of lithium daily who was admitted medically for emesis and altered mentation. Her serum lithium level was 0.5 mmol/L, but the red blood cell (RBC) lithium level was elevated at 1.0 mmol/L (therapeutic range of 0.6-1.2mmol/L). RBC lithium reflects intracellular concentrations, whereas serum lithium reflects only the extracellular compartment. The high RBC-to-serum ratio in that case suggested significant intracellular accumulation despite an apparently safe serum level [4]. A third case described a 54-year-old woman with altered mental status and involuntary movements with only a mildly elevated lithium concentration of 1.50 mmol/L (therapeutic range of 0.8-1.2 mmol/L), who required hemodialysis and was ultimately found to have nephrogenic diabetes insipidus [5]. Together, these cases illustrate that in patients with chronic lithium use, pre-existing tissue stores and the slow rate of CNS redistribution can produce severe neurotoxicity even when serum levels appear therapeutic or subtherapeutic.

## Case presentation

This case follows a 43-year-old male with a reported history of bipolar I disorder and alcohol use disorder. His mother brought him to the psychiatric emergency room three days after an intentional lithium overdose. He self-reported taking 30 to 40 300mg lithium capsules at once and did not inform anyone until three days after the overdose. The only symptom he reported was feeling “shaky and out of it.” There was no visible distress at the time of initial presentation. Labs were obtained in the emergency room, which were remarkable for a lithium level of 3.7 L/mEq (therapeutic range 0.6-1.2L/mEq) and creatinine of 1.55 (reference range 0.7-1.3mg/dL). The blood alcohol level was not detectable, and urine drug screen was negative for all illicit substances. The patient was recently admitted to complete a 28-day substance rehabilitation program and subsequently relapsed, with his last drink being five days prior to presentation. He was admitted medically to monitor, given the elevation in his serum lithium level and renal impairment. 

Once admitted to the medical floor, nephrology and psychiatry were consulted for evaluation and management. The patient began endorsing nausea, vomiting, and dizziness and was subsequently placed on an alcohol withdrawal protocol and treated with IV fluids. Serum lithium levels and renal function labs were obtained every three hours. Serum lithium continued to downtrend and normalized to 1.1 L/mEq by the next evening. His creatinine also improved with IV fluids and normalized to 1.13 mg/dL by the next evening. Nephrology did not recommend dialysis at that time. 

The patient became increasingly altered and tremulous as days progressed. Alcohol withdrawal was the primary differential for his worsening mental status, given his documented history of alcohol use disorder, recent relapse, and admission timeline of five days since his last drink. His symptoms of agitation, tremor, and altered sensorium overlapped significantly with those of alcohol withdrawal. He was receiving frequent doses of lorazepam to treat suspected alcohol withdrawal symptoms with no improvement. Phenobarbital was also initiated, given suspicion of complicated alcohol withdrawal. The patient became agitated, restless, and tried to consistently pull out his peripheral IV. He was unable to participate in conversation or follow directions, given his altered mental state. He was placed in soft restraints due to concern for his and others' safety. The patient later displayed agonal breathing, tachypnea, and extreme stupor. The patient was upgraded to the medical ICU and intubated. At this time, there was concern that despite his down-trending serum lithium level (undetectable at this point in time at <0.1 L/mEq), intracellular levels in the CNS may have remained elevated. Due to this concern, nephrology was urgently re-consulted, and dialysis was initiated. The patient received two rounds of hemodialysis over a two-day period. After the second round of dialysis, he continued to remain intubated and sedated for four days. After the patient was extubated, he remained altered and disoriented. A third round of dialysis was initiated with complete return to baseline mentation afterwards. He was fully oriented, able to follow commands, and conversational. Hospitalization was otherwise complicated by hypercalcemia treated with IV fluids and a urinary tract infection (UTI) which was treated with antibiotics. While UTI-associated delirium was considered, it was deemed unlikely to account for the severity or trajectory of his neurologic decline, as his altered mental status preceded identification of the infection and failed to improve with antibiotic treatment alone. His complete recovery following hemodialysis supported lithium neurotoxicity as the primary etiology. Upon medical stability, the patient was transferred to the inpatient psychiatric unit for further evaluation and treatment of ongoing depression and suicidality (Table 1). 

**Table 1:** Clinical Timeline BAL: blood alcohol level; CNS: central nervous system; Cr: creatinine; HD: hemodialysis; ICU: intensive care unit; Li: serum lithium; UTI: urinary tract infection. Therapeutic range: Li 0.6–1.2 mEq/L; Cr 0.7–1.3 mg/dL.

Hospital Day	Serum Lithium (mEq/L)/Creatinine (mg/dL)	Clinical Status/Symptoms	Interventions
Day 0 (ED Presentation)	Li 3.7 / Cr 1.55	Mild tremor, “shaky and out of it”; BAL undetectable; no acute distress	Admitted to the medical floor; nephrology and psychiatry consulted
Day 1	Li 1.1 (normalized)/Cr 1.13 (normalized)	Nausea, vomiting, dizziness; placed on alcohol withdrawal protocol	IV fluids; q3h lithium and renal labs; dialysis not recommended by nephrology
Days 2–3	Li downtrending (specific values not recorded)	Progressive agitation, tremor, altered mental status; unresponsive to lorazepam	Lorazepam (frequent doses); phenobarbital initiated for suspected complicated alcohol withdrawal; soft restraints
Day 4 (ICU Transfer)	Li <0.1 (undetectable)	Agonal breathing, tachypnea, extreme stupor; concern for CNS lithium toxicity despite undetectable serum level	ICU transfer; intubation; nephrology urgently re-consulted; hemodialysis initiated (HD #1 and #2 over two days)
Days 5–8	Li <0.1 (undetectable)	Remains intubated and sedated post-HD #2; altered and disoriented after extubation	Continued intubation/sedation; IV fluids for hypercalcemia; antibiotics for UTI
Day 9 (HD #3)	Li <0.1 (undetectable)	Persistent disorientation despite extubation	Hemodialysis #3 initiated
Post-HD #3	Li <0.1 (undetectable)	Complete return to baseline; fully oriented, conversational, following commands	Transferred to inpatient psychiatry for depression and suicidality

## Discussion

This case was unique, given the complexities of this patient’s initial symptoms: mild disorientation and tremors, which later evolved into dizziness, nausea, and vomiting. It is important to note that although the lithium and creatine levels were elevated upon admission, neither exceeded the limit that would necessitate immediate hemodialysis; therefore, he was subsequently managed with IV fluids and trending lithium and renal labs. He also did not display abnormal neurologic signs on admission. This treatment led to a normalization of serum lithium and creatinine values; however, he continued to decline. Given his symptoms of tremors, agitation, and restlessness, the concern for his altered mental status was protracted alcohol withdrawal and was treated as such. Phenobarbital and intermittent doses of lorazepam were administered, eventually leading to worsening confusion and state of stupor, and unresponsiveness. This acute decline resulted in intubation and three rounds of hemodialysis with a return to normal function and mental status.

In acute or chronic toxicity, serum lithium levels can be seen in the therapeutic range while the patient may still display a variety of CNS complications consistent with lithium toxicity. This unique presentation can be difficult to diagnose to the untrained eye and can subsequently cause a possible delay in treatment. The Extracorporeal Treatment in Poisoning (EXTRIP) workgroup is a committee of international experts of various medical specialties who create evidence-based guidelines for toxicities that require extracorporeal treatment [6]. The EXTRIP criteria for lithium toxicity suggest hemodialysis for patients with significant confusion, a serum level greater than 5.0 mEq/L (therapeutic range: 0.6-1.2mEq/L), or if the estimated time to reduce the serum lithium concentration to less than 1.0mEq/L is greater than 36 hours [6]. They also recommend hemodialysis for a lithium level greater than 4.0 mEq/L with clinical signs of renal impairment or if the patient is experiencing seizures, life-threatening dysrhythmias, or a decreased level of consciousness. These signs of renal impairment are further defined as: the presence of oligo/anuria, stage 3B, 4, or 5 CKD, stage 2 or 3 AKI, or serum creatinine levels of 2 mg/dL or 1.5 mg/dL (reference range: 0.6-1.3 mg/dL) in adults and elderly/low muscle mass patients, respectively [6]. This patient’s serum levels did not meet these criteria at any point of his hospitalization despite a neurologic decline in the setting of therapeutic and subtherapeutic serum lithium levels. The presence of neurologic decline was likely related to the difference in the rate of lithium elimination between the serum and the central nervous system. In the case of an acute overdose without prior lithium use, serum lithium levels correlate more with the severity of clinical manifestations because there are no pre-existing stores in the tissues [1]. This results in a faster renal clearance of the drug from the body. However, in acute-on-chronic presentations, neurologic symptoms may be delayed or prolonged despite down-trending serum levels due to the redistribution of prior lithium stores in tissues and the CNS to the extracellular fluid via passive diffusion [1]. These tissue reservoirs and the slow rate of passive diffusion make trending serum lithium a less reliable indicator of the severity of toxicity in acute-on-chronic cases. It is also important to note that the redistribution in tissues is also responsible for a possible rebound and continued elevation in serum lithium levels and return of clinical manifestations 6-24 hours after hemodialysis management [6]. This would result in additional hemodialysis treatments with trending labs to monitor for any ensuing rebounds.

## Conclusions

The error made in this case was misdiagnosing the patient’s altered mental status and agitation as alcohol withdrawal when in fact the patient was suffering from lithium toxicity in the setting of subtherapeutic and eventually nondetectable serum lithium. This resulted in the patient being inappropriately treated with high doses of lorazepam and phenobarbital, which likely worsened his mentation. Additionally, it resulted in a serious delay of treatment with hemodialysis. It is important to note from this case that there could be high discrepancy between intracellular and extracellular levels of lithium. As a solution, stricter guidelines may need to be in place when treating lithium overdose and toxicity, whether acute or chronic. Hemodialysis may need to be initiated more liberally when there are several confounding factors, particularly in patients with chronic lithium use presenting with progressive neurologic symptoms despite declining serum levels. This recommendation is drawn from a single case and should be interpreted cautiously; prospective validation and comparison against established guidelines such as the EXTRIP criteria are needed before broader changes to practice can be advocated. Monitoring serum lithium is important; however, assessing clinical presentation is also imperative.
